# Metal-Organic Framework Materials for Perovskite Solar Cells

**DOI:** 10.3390/polym12092061

**Published:** 2020-09-10

**Authors:** Do Yeon Heo, Ha Huu Do, Sang Hyun Ahn, Soo Young Kim

**Affiliations:** 1 Department of Materials Science and Engineering, Korea University, 145 Anam-ro, Seongbuk-gu, Seoul 02841, Korea; doyoun0312@naver.com; 2 School of Chemical Engineering and Materials Science, Chung-Ang University, 84 Heukseok-ro, Dongjak-gu, Seoul 06974, Korea; hadohuu1311@gmail.com

**Keywords:** metal-organic frameworks, MOF, solar cell, perovskite solar cells

## Abstract

Metal-organic frameworks (MOFs) and MOF-derived materials have been used for several applications, such as hydrogen storage and separation, catalysis, and drug delivery, owing to them having a significantly large surface area and open pore structure. In recent years, MOFs have also been applied to thin-film solar cells, and attractive results have been obtained. In perovskite solar cells (PSCs), the MOF materials are used in the form of an additive for electron and hole transport layers, interlayer, and hybrid perovskite/MOF. MOFs have the potential to be used as a material for obtaining PSCs with high efficiency and stability. In this study, we briefly explain the synthesis of MOFs and the performance of organic and dye-sensitized solar cells with MOFs. Furthermore, we provide a detailed overview on the performance of the most recently reported PSCs using MOFs.

## 1. Introduction

### 1.1. Metal-Organic Frameworks

**Figure 1:**
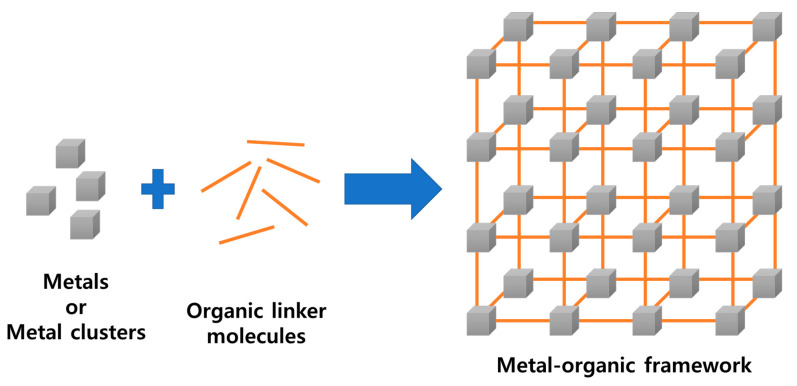
Schematic of the metal-organic framework (MOF) structure.

Porous materials are widely used in various fields owing to their large specific surface area and easy and fast diffusion of ions and electrons through the pores. Porous materials include porous ceramics, polymer foams, porous glass, activated carbon, porous metals, and zeolites; among these materials, metal-organic frameworks (MOFs) have recently attracted attention. MOF is a porous, organic–inorganic hybrid compound, wherein a metal ion and an organic ligand are connected by coordination to form a three-dimensional structure (Figure 1).

In MOFs, transition metals, actinides, alkaline earth metals, and mixed metals are primarily employed as the inorganic metals, whereas carboxylates, sulfates, phosphonates, azoles, and heterocyclic compounds are commonly used as organic linkers. Because MOFs have a significantly large surface area and open pore structure, they can transport larger amounts of molecules or solvents compared to other porous materials. Another advantage of MOFs is their ability to modify the composition of the metal-organic ligands, thereby showing various properties and controlling the size of pores. For these reasons, MOFs have been used in a wide range of applications, such as hydrogen storage and separation [1,2,3,4,5], catalysis [6,7,8], and drug delivery [9,10].

Investigations based on MOFs started in 1990s [11,12,13,14,15], and the term MOF was first introduced in 1995 [13]. Since then, studies based on MOFs have been actively conducted, and the interest in MOFs increased significantly when MOF-derived materials were introduced by Hu et al. [16]. MOF-derived materials can comprise various hybrid materials because they can derive materials by changing the MOF precursors, guest species, and synthetic conditions. MOF-derived materials, characterized by high surface area, porosity, and improved structural stability, have been studied as a variety of catalysts [17,18]. MOFs and MOF-derived materials with high structural variability can be applied to energy-related processes and to numerous other fields. With the recent rapid development in the photovoltaic technology, researches based on the utilization of MOFs in energy applications has garnered considerable attention. Herein, we summarize the synthesis methods for MOFs and their use in solar cells. In particular, recent studies on the application of MOFs to perovskite solar cells (PSCs), which are highlighted as next-generation solar cells that may replace silicon solar cells, are summarized.

### 1.2. Synthesis of MOFs

**Figure 2:**
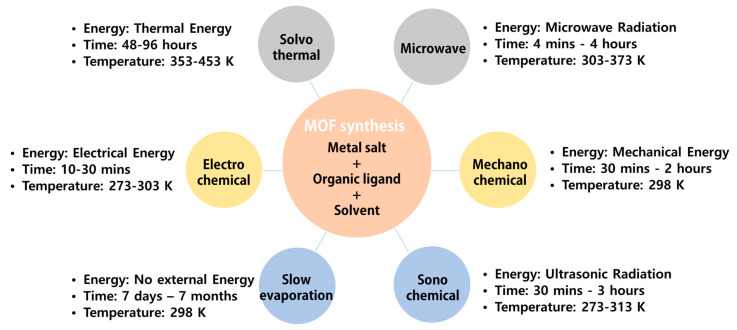
Summary of various MOF synthesis methods.

Most MOF syntheses are performed in the liquid state. The metal salt and ligand solvent are separately prepared and then mixed or the metal salt and ligand are added to the solvent. In general, organic solvents with high solubility, such as dimethylformamide, acetonitrile, acetone, diethyl formamide, ethanol, and methanol, are used. Since the solvent affects the properties of MOFs, it must be selected according to the desired characteristics of the resultant MOFs. The synthesis method can be determined according to the solvent, the characteristics of the MOF, the required pore and particle size of the MOF, and the laboratory conditions. The methods for synthesizing MOFs are summarized in Figure 2.

#### 1.2.1. Solvothermal Synthesis

Solvothermal methods are the most common techniques for synthesizing MOFs with various morphologies. Organic solvents or a mixture of metal salt solutions are reacted with organic ligands. Solvothermal synthesis is performed at a temperature higher than the boiling point of the solvent, and a relatively high yield of MOF materials can be obtained. The solvothermal route is advantageous for precisely controlling the distribution of morphologies, crystallinity, and the size of the produced materials. The type of solvent, synthesis temperature, concentration of reactants, and pH of the solution should be carefully selected. Pachfule et al. succeeded in synthesizing two structurally different two-dimensional (2D) fluorinated MOFs by the solvothermal reaction of Cu(NO_3_)_2_·3H_2_O with 4,4′-(hexafluoroisopropylidene)bis(benzoic acid) (C_17_H_10_F_6_O_4_, H_2_hfbba) and terminal monodentate ligand 3-methyl pyridine (3-picoline/3-mepy) in the presence of *N*,*N*-dimethylformamide (DMF) and *N*,*N*-diethylformamide (DEF) solvents [19]. The effect of solvent selection was reflected in the synthesized MOF structure. The authors found that the different solvents employed for the polymer backbone resulted in various changes in the material due to different degrees of deprotonation of H_2_hfbba under appropriate conditions. The Yang group studied zeolitic-imidazole frameworks-78 (ZIF-78), which have an anisotropic pore structure and an affinity for CO_2_, using solvothermal synthesis to control the morphology [20]. They produced hexagonal rod-shaped ZIF-78 microcrystals using triethylamine. They also succeeded in adjusting the size and aspect ratio of the ZIF-78 microrods from “slender” to “squat” by altering the nutrient and ligand concentration and the relative molar ratio of 2-nitroimidazole to 5-nitrobenzimidazole. Recently, Wang et al. succeeded in synthesizing flower-string-like NiCo–MOF/multiwall carbon nanotube (MWCNT) complexes through a simple solvothermal method [21]. MWCNTs were used as a substrate for bimetallic NiCo–MOF growth, whereas 4,4′-biphenyldicarboxylic acid was used as a ligand. The authors confirmed that the presence of MWCNTs did not affect the formation of NiCo–MOF crystals and can serve as a guide for the growth of MOF. Several well-dispersed flower-string-like structures were obtained by controlling the added amount of MWCNTs. The solvothermal method is disadvantageous because it is a complex process that requires the removal of solvent molecules from the pores. In general, this is accomplished through vacuum drying or washing with a solvent such as ethanol or methanol.

#### 1.2.2. Microwave-Assisted Synthesis

Microwave-assisted synthesis is widely used as a quick and simple method to generate MOFs. The driving force of this synthesis is microwave power. This synthetic method has a low reaction time and produces highly crystalline and porous textures and allows precise shape control and particle size reduction. Ni et al. synthesized MOFs in 30 to 120 s using a microwave-assisted technique, which previously took several hours or days [22]. They also increased the yield from 30% to over 90%. The authors changed the size of the crystals by controlling the concentration of the reactants. Since all the crystals nucleated simultaneously, the nucleation process was controlled, resulting in small-sized crystals. Vakili et al. successfully synthesized zirconium-based MOFs using microwave-assisted synthesis and optimized the yield and porosity of the MOFs by regulating the amounts of modulators (i.e., benzoic acid and hydrochloric acid), temperature, and time [23].

#### 1.2.3. Slow Evaporation Method

The slow evaporation method produces MOFs by gradually concentrating a precursor dissolved in a solvent or mixture of solvents by slow evaporation in an inert atmosphere. Slow evaporation method requires 7 days to 7 months of synthesis time without external energy. Utilizing a mixture of solvents increases the solubility. This technique is appealing because the MOFs can be synthesized without supplying external energy; however, it is a time-consuming process. Cui et al. synthesized MOFs using a spherical colloidal crystal as a three-dimensionally aligned host matrix by slow evaporation [24]. The fabricated MOF-optical colloidal crystal exhibited interesting optical properties, specific molecular recognition, anisotropy, and derivatization ability. Its unique optical bandgap facilitated the manipulation of photophysical and photochemical behaviors.

#### 1.2.4. Mechanochemical Synthesis

Mechanochemical synthesis causes a chemical reaction that occurs due to the mechanical agitation and impact between materials, and the reaction can proceed without the use of carcinogenic, toxic, or environmentally harmful organic solvents. The starting materials are generally metal oxides instead of metal salts. Since no solvent is employed, there is no need to heat, thereby saving energy. In addition, highly crystalline materials can be obtained with this simple process. The Li group proposed a mechanochemical method to rapidly synthesize MOF-5 with a high Brunauer–Emmett–Teller (BET) area [25]. They confirmed that solvent activation had a significant effect on the formation of MOF-5. MOF-5-B optimized by mechanochemical synthesis showed preferential adsorption for long chain alkanes at low pressure. MOF-505 was synthesized using the mechanochemical technique, and it was confirmed that the type and amount of the added solvent were decisive factors in producing MOF-505 [26].

#### 1.2.5. Sonochemical Synthesis

This is a method for synthesizing MOFs by utilizing ultrasonic waves (20–10 MHz). When the reaction mixture is exposed to ultrasonic waves, the molecules are chemically altered to produce compounds with new morphologies and unique properties. The ultrasonic waves also cause physical transformation, including the formation and growth of small bubbles in the liquid phase. The collapse of these bubbles creates short-lived local hot spots at high temperatures and pressures, resulting in uniform nucleation. Moreover, sonochemical synthesis can reduce the crystallization time compared to the conventional solvothermal synthesis. In 2008, the Ahn group succeeded in producing MOF-5 crystals for the first time using the sonochemical method [27]. MOF-5 crystals synthesized by this technique were found to have similar properties to crystals prepared with conventional convective heating; however, the synthesis time was shorter and considerably smaller sized crystals were generated. The same group successfully prepared Mg-MOF-74 crystals in 1 h using triethylamine as a deprotonating agent [28], and the Mg-MOF-74 showed excellent selectivity to CO_2_ and exceptional catalytic performance.

#### 1.2.6. Electrochemical Synthesis

Since electrochemical synthesis does not require a metal salt, the anion associated with the metal salt is not present; therefore, a high purity substance can be obtained. In this method, metal ions are supplied through the oxidation of electrodes. When an appropriate voltage or current is applied, the metal dissolves and the metal ions necessary for MOF formation are released from the electrode surface. These metal ions immediately react with the linker present in the solution and MOF is formed in proximity to the electrode surface; in this way, the film cracking is reduced. Campagnol et al. first synthesized MIL-100(Fe) using the electrochemical synthetic method [29]. This technique paved the way for the production and deposition of a variety of MOFs that cannot be synthesized at low temperatures, such as MIL-100(Fe), since the reaction occurs under milder conditions. MOF-5 was electrochemically synthesized by Yang et al. in a 1-butyl-3-methylimidazoliume bromine ion liquid system using zinc nitrate as a partial metal source and terephthalic acid as an organic ligand [30]. The coordination effect between 1,4-benzene-dicarboxylate and Zn^2+^ induces the crystallization of the MOF-5 and forms an infinite network depending on the template composition.

Other ways to synthesize MOFs include the diffusion method, spray drying, and ionothermal synthesis [31]. The diffusion method can be divided into the gas phase, liquid phase, and gel diffusion, but the solvents used are different. In the gas phase diffusion method, a volatile organic ligand solution is used as a solvent, whereas in the liquid phase diffusion method, organic ligands and metal ions are dissolved in immiscible solvents. The synthesis of MOF crystals by gel diffusion occurs in a mixture of metal ion solutions and organic ligands dispersed in a gel material. The diffusion method is performed under mild reaction conditions; however, the reaction time is long. The spray-drying method involves spraying the MOF precursor solution using a post nozzle to generate a fine droplet spray. When the solvent evaporates, the precursor concentration on the surface increases and crystallization proceeds until a critical concentration is reached. The ionothermal method can control the physical and chemical properties of the MOF by changing the composition of the ionic liquid employed as the structural template, reaction medium, and charge balance group.

## 2. MOFs in Solar Cells

### 2.1. Application to Solar Cells

A solar cell converts light energy from the sun into electrical energy to generate electricity. Solar cells are environmentally friendly because they do not emit harmful substances when electricity is produced, unlike nuclear power plants and coal fuel. Solar cells operate on the basis of the photovoltaic effect, wherein voltage and current are generated when the surface of the cell is irradiated. The photovoltaic effect is when carriers (electrons or holes) become excited inside a material to generate voltage or current. Classical solar cells comprise a semiconductor material, with a bandgap smaller than the energy of visible light, which can absorb light. This light excites electrons to the conduction band, and the excited electrons flow as current due to an externally applied voltage. The external voltage is created by joining p- and n-type semiconductors. In this case, when band bending occurs at the interface of the two semiconductors due to a difference in the Fermi energies, an electric field is generated between the p- and n-type semiconductors. That is, the electrons excited by irradiated light to the conduction band can move from the p- to n-type semiconductor due to band bending of the p–n junction, thereby generating an electric current that flows along the circuit to be used as energy.

**Figure 3:**
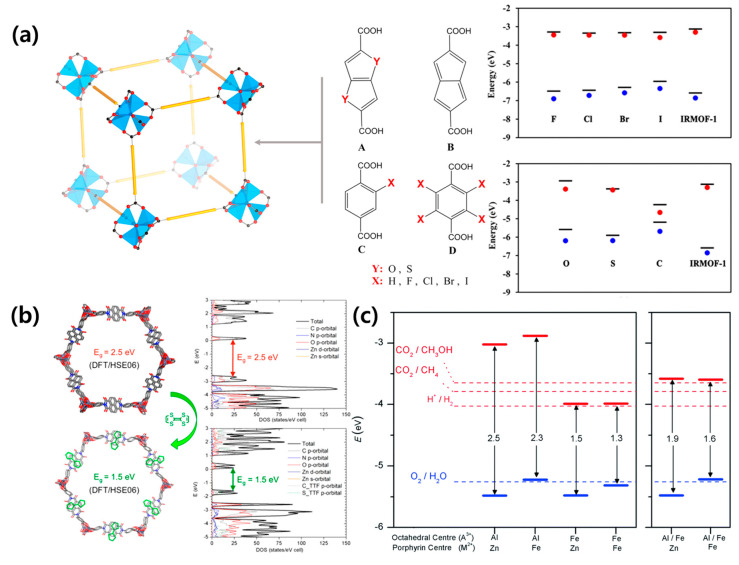
(**a**) Schematic of the MOF and organic linkers (left) and band edge positions of the MOFs with various linkers (right). Reprinted from [38] with permission. Copyright© 2014, American Chemical Society. (**b**) Simulated structures (left) and the corresponding band structures (right) of DSNDI-based MOF-74. Upon TTF intercalation, the bandgap of the MOF drops from 2.5 to 1.5 eV. Reprinted from [39] with permission. Copyright© 2017, American Chemical Society. (**c**) Bandgaps and band edge positions, as calculated with the screened hybrid functional HSE06 for (left) porphyrin-based MOF with only one element (A = Al or Fe) at the octahedral sites, and (right) mixed perovskite-MOF (P-MOF) with 50% Al and 50% Fe at the octahedral sites. Reprinted from [40] with permission. Copyright© 2017, Royal Society of Chemistry.

The variables that determine the efficiency of a solar cell are the open-circuit voltage (*V_OC_*), short-circuit current (*J_SC_*), fill factor (FF), and power conversion efficiency (PCE). *V_OC_* is the potential difference formed at both ends of a solar cell upon light irradiation when the circuit is open, i.e., an infinite resistance. The *V_OC_* is determined by the bandgap of the semiconductor; a high *V_OC_* is generally obtained when a material with a large bandgap is used. *J_SC_* is defined as the current density in the reverse direction (negative value) upon the illumination of cell with light when the circuit is shorted, i.e., with no external resistance. The *J_SC_* depends on the intensity of the incident light and spectral distribution. However, when these conditions are known, *J_SC_* depends on how effectively the electrons and holes that are generated by light absorption are recombined and not lost, and how effectively they are transported from the cell to the external circuit. Losses due to recombination can occur inside or at interfaces in the cell [32,33,34]. A high *J_SC_* can be obtained by maximizing the reflection of sunlight on the surface of the solar cell. Consequently, metal contacts that minimize the area that blocks the sunlight [35] or antireflection coatings are applied [36,37]. A small semiconductor bandgap energy is advantageous to absorb light of all possible wavelengths. However, if the bandgap is small, *V_OC_* decreases; therefore, a material possessing an appropriate bandgap is required. The theoretically optimal bandgap energy to maximize *V_OC_* and *J_SC_* is 1.4 eV. FF is a value that indicates how close the current density–voltage (*J–V*) curve is to a rectangle when light is applied. The PCE is defined as the value of solar power output divided by the solar radiation per area. FF and PCE can be determined using the following formulas:

(1) $$FF=\;\frac{V_{MP}\times J_{MP}}{V_{OC}\times J_{SC}},$$

(2) $$PCE=\;\frac{V_{OC}\times J_{SC}\times FF}{P_{input\;}}\times 100,$$

where *V_MP_* and *J_MP_* are the voltage and current density values at the maximum power point, respectively. As mentioned earlier, it is important to use a material with an appropriate bandgap to absorb light. The bandgap of MOFs comprising a metal ion and organic linker can be easily controlled by changing the constituent components. The electronic band structure of isoreticular MOFs was investigated by density function theory [38]. A halogen atom can be used as a functional group and affords control of the bandgap and valence band maximum (VBM) (Figure 3a). It was found that iodine is the best candidate to reduce the bandgap and increase the VBM. Guo et al. designed a new naphthalenediimide ligand-based MOF-74 prepared with two salicylic acid groups (DSNDI) [39]. The authors succeeded in adjusting the bandgap of the MOF from 2.5 to 1.5 eV through the intercalation of tetrathiafulvalene (TTF) to DSNDI-based MOF-74 (Figure 3b).

Due to charge delocalization throughout the electron and donor/acceptor stack injected by the TTF guest molecule, the conduction band of TTF-doped MOF remains near the Fermi level, and the valence band significantly shifts upwards, thereby reducing the bandgap. In addition to halogen ion and TTF doping, the effect of Fe substitution on the bandgap of porphyrin-based MOF was studied [40]. The authors confirmed that the presence of Fe in the porphyrin metal center slightly increases the position of valence band edge, whereas Fe at the octahedral metal node can significantly lower the position of the conduction band edge (Figure 3c). Therefore, Fe is a useful dopant material for regulating the band structure and alignment in MOFs. Furthermore, the bandgap can be adjusted according to the dopant, metal, and organic linkers, thereby making it a suitable material for solar cells [41,42,43].

Solar cells can be classified according to their constitution and type of materials used. The most widely known solar cells are based on silicon. These can be divided into monocrystalline, polycrystalline, and amorphous silicon solar cells depending on the crystallinity of silicon. Among these solar cells, single-crystal silicon solar cells exhibit the highest light conversion efficiency, although the fabrication cost associated with these cells is high. Such solar cells are referred to as first-generation solar cells [44]. Second-generation solar cells concern thin-film constructions. The compound semiconductor copper indium gallium selenide (CIGS) is typically studied in this type of solar cell [45]. A CIGS thin-film solar cell possesses a higher light absorption rate than crystalline silicon, thus it is possible to manufacture high-efficiency cells with a thickness of 1–2 μm. Furthermore, the production process is simpler than that of a silicon solar cell, thereby reducing cost. Third-generation solar cells are based on the second generation solar cells, with new materials such as organic semiconductors employed as the active layer. Using organic semiconductors, the process temperature can be lowered and the choice of substrate can be widened. Since a solar cell can also be developed on a flexible substrate, it is possible to produce a curved solar cell that can be applied in various fields. Herein, we discuss third-generation solar cells, wherein MOFs are utilized.

### 2.2. Organic Solar Cells with MOFs

Organic solar cells (OSCs) have the advantages of highly tunable structures, low-cost, large-area manufacturing, flexibility, and translucent devices; however, they lack high PCEs. Therefore, several studies have been conducted to improve the PCE and stability of OSCs [46,47,48,49,50,51,52,53,54,55,56,57,58]. In OSCs, the interfacial layers, including the electron extraction layer (EEL) and hole extraction layer, play a critical role, and high conductivity and charge transport mobilities are prerequisites for the interfacial layers. Two-dimensional materials are ideal for use as an additive to the interfacial layer due to their large surface areas and excellent electronic and optical properties. In particular, transition metal dichalcogenides (TMD) have attracted considerable attention owing to their exciting optoelectronic properties, bipolar charge transport, chemical stability, and direct bandgap in a single layer form [59]. However, it is difficult to control the thickness and scalable production of TMDs, which limit their application in OSCs. Therefore, two-dimensional MOFs that also possess the excellent properties of TMDs are extremely attractive materials for solar cells. Xing et al. [60] proposed an effective method for peeling the tellurophene-based 2D MOF and branched surfactant polymer polyethylenimine ethoxylate (PEIE). They also succeeded in improving the PCE of OSCs using PEIE-functionalized MOF nanosheets as an interlayer. The reason for improved photovoltaic performance is the numerous characteristics such as the tunable work function of the PEIE–MOF interfacial layer, improved conductivity, and passivation of oxygen defects on the ZnO film (as an EEL). Recently, Sasitharan et al. synthesized ultrathin zinc porphyrin-based MOF nanosheets (MONs) [61]. The OSCs employing MONs as the photoactive layer showed a PCE of 5.2%, which is almost twice as much as the reference device. Owing to their electronic, optical, and structural properties, MONs afforded a surface template for the crystallization of poly(3-hexylthiophene-2,5diyl) (P3HT). Therefore, increasing the absorbance two times, increasing the hole mobility, and reducing the grain size led to improved PCEs. These results demonstrate the potential of tunable 2D MOF nanosheets as materials to improve the performance of a wide range of OSCs.

### 2.3. Dye-sensitized Solar Cells with MOFs

Dye-sensitized solar cells (DSSCs) are cost-effective because they are manufactured with inexpensive materials and have simple manufacturing processes. DSSCs are composed of titanium dioxide (TiO_2_) and a dye sensitizer that can be extracted from natural resources. Graphene can be used as an electrode, replacing the expensive Pt metal [62,63]. DSSCs can be fabricated using a roll-to-roll process; a continuous and inexpensive method of printing on flexible substrates. DSSCs can be assembled in various locations such as windows and sunroofs because they can function effectively using diffused light even cloudy weather. Owing to these advantages, there has been intense research on DSSCs [64,65,66,67,68,69].

Despite significant efforts to improve the *J_SC_* and PCE in DSSCs, the *V_OC_* remains relatively low due to charge recombination at the TiO_2_/dye and TiO_2_/electrolyte interfaces. MOFs contain nanosized channels and cavities with a BET surface area greater than 5000 m^2^ g^−1^ [70]. Therefore, the facile fabrication and high porosity of MOFs make them suitable materials for improving the performance of DSSCs. The Wei group first used ZIF-8 MOFs to coat the TiO_2_ electrode in DSSCs [71]. They used ZIF-8 and observed a linear relationship between the thickness of the ZIF-8 coating layer and the value of *V_OC_*. Additionally, the ZIF-8 shell material is due to the inhibition of interfacial charge recombination and helps to improve the *V_OC_*. These results proved to be the starting point for the study of MOFs in DSSCs.

**Figure 4:**
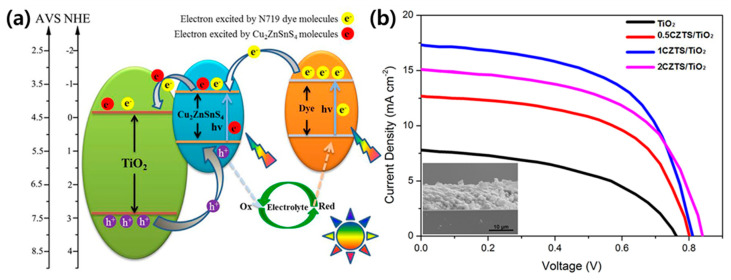
(**a**) Energy band structure and photogenerated charge transfer mechanism in Cu_2_ZnSnS_4_ (CZTS) nanoparticles/MOF-derived TiO_2_. (**b**) Current density–voltage curves of MOF-derived TiO_2_ and CZTS/TiO_2_ samples. Reprinted from [72] with permission. Copyright© 2016, American Chemical Society.

MOFs can be applied in DSSCs as the photoanode, counter electrode, and electrolyte. Tang et al. synthesized Cu_2_ZnSnS_4_ (CZTS) nanoparticle-sensitized MOF-derived mesoporous TiO_2_ and employed it as a photoanode in DSSCs [72]. MOF-derived TiO_2_ inherits the large specific surface area and rich porous structure of the parent MOF. Therefore, the dye loading ability is improved, multiparticle light scattering process is enhanced, and incident light propagation length is extended. The heterostructure formed between the CZTS nanoparticles and MOFs-derived TiO_2_ effectively prolongs the carrier lifetime and suppresses the electron/hole recombination rate due to the matched bandgap (Figure 4a). Compared to pure TiO_2_, the specific surface area of CZTS/TiO_2_ is substantially reduced, and the micropores disappear almost immediately after the deposition of CZTS nanoparticles. The CZTS/MOF-derived TiO_2_-based DSSC displayed a maximum photocurrent of 17.27 mA/cm^2^ and a photoelectric conversion performance of 8.10%, approximately two times higher than the TiO_2_-based DSSCs (Figure 4b). The porous structure and large surface area resulting from the introduction of the MOFs is believed to enhance the performance of DSSCs by augmenting the transport of interfacial carriers, dye adsorption, and light harvesting.

Carbon materials [73,74], transition metal materials [75,76,77], metal alloys [78], and conductive polymers [79] have been developed to replace the Pt counter electrode in DSSCs. Carbon materials are promising metal-free electrode materials with high surface area and electrical conductivity; however, their stability is relatively poor. To compensate for the shortcomings of carbon materials, Ou et al. synthesized a transparent CoS_1.097_@N-doped carbon film derived from a cobalt-metalloporphyrin MOF thin film and used it as a counter electrode in DSSCs [80]. The CoS_1.097_ nanoparticles were uniformly dispersed on the N-doped carbon film, facilitating electron transfer. CoS_1.097_@N-doped carbon films presented higher PCEs of 9.11% and 6.64% (front and rear irradiation, respectively), compared to Pt (8.04% and 5.87%). They also showed excellent long-term stability over 1000 h under ambient conditions, indicating their superiority to the Pt counter electrode.

MOFs can be used as an additive to the electrolytes in DSSCs. Bella et al. dispersed an Mg-MOF in a polymer matrix using a UV-induced free-radical process and employed the resultant material as a polymer electrolyte in quasi-solid DSSCs [81]. The interaction between the organic shell of the Mg-MOF particles and surface group of the TiO_2_ layer allowed DSSCs with a PCE of 4.8% and long-term durability to be fabricated. Table 1 lists the photovoltaic performances of MOFs in DSSCs.

The use of MOFs in quantum dot-sensitized solar cells (QDSSCs) has also attracted research attention [92,93,94,95,96]. QDs are employed as a solid-state sensitizer and have advantages such as quantum attenuation, wide light absorption, and multiple exciton generation. MOFs can also behave as photosensitizers owing to their chemical and physical properties, porosity, and long-range internal energy transport paths. Kaur et al. demonstrated a CdTe QD/europium-MOF (Eu-MOF) complex with photocatalytic properties as a photoanode in QDSSCs [97]. The CdTe/Eu-MOF QDSSC showed improved PCE (3.02%) compared to CdTe QDSSC (PCE = 1.67%). These results suggest that appropriate mixing of QDs with MOFs can improve the light-harvesting capacity in QDSSCs. The potential of ZIF-67 as a counter electrode was studied by Xu et al. [98]. Compared to the PCE of CdSe QDSSCs using a Pt counter electrode (2.98%), QDSSC with a ZIF-67 counter electrode exhibited a photoelectric conversion efficiency of 3.77%. This highlights the possibility that MOF can be applied as a counter electrode of QDSSC in place of Pt.

**Figure 5:**
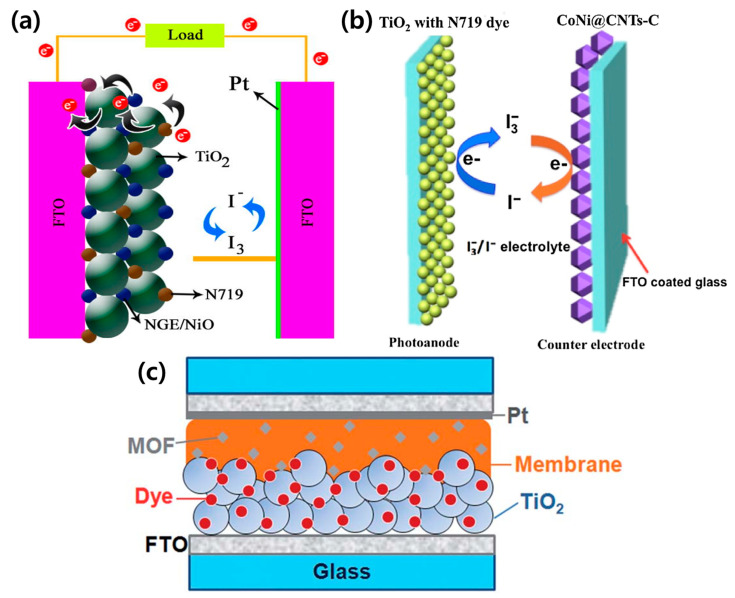
Schematic of a DSSC configuration using MOFs as the (**a**) photoanode [85], (**b**) counter electrode [90], and (**c**) electrolyte [81]. Reproduced form [81] with permission from The Royal Society of Chemistry. Reprinted from [85] with permission. Copyright© 2017, Elsevier. Reprinted from [90] with permission. Copyright© 2017, Elsevier.

Recently, MOF researchers, as well as photovoltaic researchers, have made great efforts to utilize MOFs in photovoltaic cells in addition to the other main application fields of MOFs, such as catalysis, sensors, and gas storage. Among the various types of photovoltaic cells, MOFs are incorporated in DSSCs by replacement of or mixing with the existing photoanode, counter electrode, and electrolyte (Figure 5).

MOFs using a photosensitive linker with a high electrical conductivity are suitable for use as the photoanode. MOF-derived materials can be applied as a counter electrode that replaces the expensive Pt electrode. MOFs as an electrolyte led to efficiency improvements and long-term durability; however, further research is required. As mentioned earlier, DSSCs have attracted significant attention owing to their low-cost and simple manufacturing process; nevertheless, the liquid electrolyte and low efficiencies hinder commercialization. The introduction of perovskites to replace the liquid electrolyte was the spark that ignited the hot topic of PSCs. Henceforth, we assess MOFs integrated into PSCs.

## 3. Perovskite Solar Cells with MOFs

**Figure 6:**
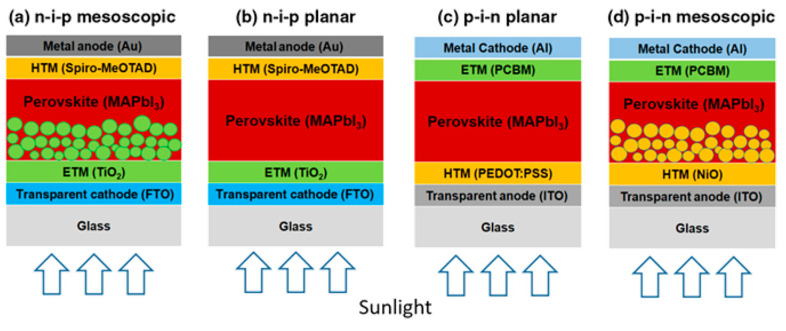
Schematic of perovskite solar cells with (**a**) n–i–p mesoscopic, (**b**) n–i–p planar, (**c**) p–i–n planar, and (**d**) p–i–n mesoscopic structures. Reprinted from [101] with permission.

The PCE of PSCs has increased rapidly from 3.8%, reported by the Miyasaka group in 2009, to up to 25.2% [99,100]. The general structural formula of perovskites is ABX_3_, where the A site is an inorganic or organic cation (Cs^+^, Rb^+^, methylammonium (MA) CH_3_NH_3_, formamidinium (FA); CH_2_(NH_2_)_2_^+^, guanidinium), the B site is a divalent metal (Pb^2+^, Sn^2+^, Bi^2+^, Ge^2+^), and the X site is a halide (I⁻, Br⁻, Cl⁻, or their mixtures; SCN⁻). Perovskites exhibit low recombination losses, low-cost processing, long charge carrier diffusion lengths, and facile bandgap tunability, making them suitable for use as a light absorber. PSCs comprise a conductive substrate (fluorine-doped tin oxide (FTO) or indium tin oxide (ITO)), electron transport layer (ETL), hole transport layer (HTL), light absorber (perovskite layer), and a metal electrode. PSCs are primarily divided into mesoporous or planar structures and can be further classified into standard (n–i–p) and inverted (p–i–n) structures, as shown in Figure 6 [101].

TiO_2_, which is mainly used in DSSCs, is also used in PSCs as an electron transport material. The mesoporous metal oxide layer functions as a scaffold to protect the perovskite film and transfer electrons from the conduction band of the perovskite to the compact TiO_2_ layer. The mesoscopic n–i–p structure is the most commonly used and high efficiencies have been obtained with this configuration. In a planar structure without the mesoporous layer, light can pass through the glass substrate to the hole transport material and improve the PCE, since the top contact electrode can be completely covered [102]. Inverted (p–i–n) structures are fabricated on a conductive substrate starting with a HTL and perovskite layer, ETL, and metal cathode are subsequently deposited. The p–i–n structure is advantageous because it decreases the process temperature compared to the n–i–p structure; however, the slightly lower efficiency is a disadvantage.

PSCs have a high PCE which is comparable to single-crystal silicon solar cells. Nevertheless, the long-term stability issues impede the commercialization of PSCs. In general, the perovskite film contains many defects and grain boundaries (where electron/hole recombination occurs); these constrain the performance of the device. Therefore, recent PSC-based researchers have focused on improving the stability as well as the efficiency [103,104]. Studies have been performed to enhance the crystallinity, using inorganic ions [105,106,107], additives [108,109,110], new ETLs or HTLs [111,112], and interlayers [113,114].

**Figure 7:**
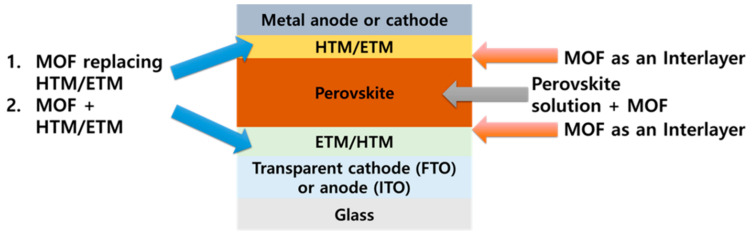
Schematic of where MOFs can be used in perovskite solar cells (PSCs).

Chemically and thermally stable nanostructured MOFs are attracting considerable attention as an appealing material for PSCs. In addition to their stability, MOFs can be solution processed through the simple solution synthesis and deposition. Furthermore, the optoelectronic properties of MOFs can be tuned by controlling the constituent metal ions and organic linkers, and they can thus be used in a variety of ways in PSCs. Figure 7 shows four applications of MOFs in the PSC structure. MOFs provide excellent electron and hole transport paths and are effective in suppressing charge recombination by improving the quality of perovskite films. We identify and explain the various roles of MOFs in PSCs.

### 3.1. MOFs as the Electron Transport Material

**Figure 8:**
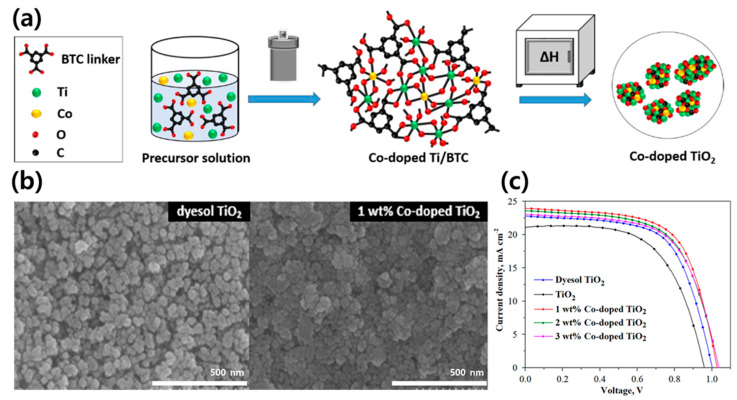
(**a**) Schematic of the preparation of Co-doped TiO_2_ particles (BTC: trimesic acid). (**b**) SEM images of dyesol TiO_2_ and 1 wt % Co-doped TiO_2_ paste on FTO-coated glass. (**c**) current density–voltage (*J*–*V*) curves of the best performing PSCs using dyesol TiO_2_, undoped TiO_2_, and Co-doped TiO_2_ (1, 2, and 3 wt %). Reprinted from [118] with permission.

Electron transport materials (ETMs) in solar cells must have high carrier mobilities and their energy levels should match the energy levels of other layers in the solar cell. High specific surface areas and low amounts of defects play important roles in improving the PCE. As mentioned above, TiO_2_ is mainly utilized as the ETM in PSCs owing to its excellent structural stability and low cost [115,116,117]. However, the bandgap of commercial TiO_2_ is approximately 3.3 eV, which is in the ultraviolet range. The large bandgap allows the excitation and injection of electrons, making electron transport inefficient. To solve this problem, it is important to reduce the bandgap, and therefore, a method to dope the semiconductor with metal has been proposed. Nguyen et al. synthesized cobalt(Co)-doped TiO_2_ MOFs using the solvothermal method [118]. Co reduces the bandgap of TiO_2_ and facilitates distortion due to the presence of Co defect atoms in the TiO_2_ lattice. Titanium(Ti)-MOF was calcinated in an ambient atmosphere to convert Ti to TiO_2_ (Figure 8a), and 1 wt% Co-doped TiO_2_ MOF presents a structure with a higher porosity than dyesol TiO_2_ and shows better photovoltaic performance (Figure 8b,c). While the PCE of dyesol TiO_2_ PSCs was 12.32%, it was confirmed that doping of the TiO_2_ MOF with Co increased the PSC efficiency to 15.75%. The charge transport resistance (R_TRANS_) and charge recombination resistance (R_REC_) significantly diminished due to the Co doping, and the dopant promotes electron transport and moderates electron–hole recombination. These results are attributed to the improvement in the electron transport resulting from internal and surface morphological rearrangements and Co doping obtained by the thermal decomposition of the MOF template.

As mentioned above, some studies have mixed MOFs and TiO_2_, whereas others have inserted the MOF layer between the TiO_2_ and perovskite layers as a mesoporous transport layer. ZIF-8 was coated on the TiO_2_ layer and the performances of the resultant PSCs were compared according to the reaction time [119]. When ZIF-8 was present, the PCE improved from 9.6% to 12.0%, and it was confirmed that the immersion time to obtain an optimal ZIF-8 coating was 2 min. The ZIF-8 MOF layer located between the TiO_2_ and perovskite layers improves the transfer rate of excited electrons and the absorbance of the perovskite film at wavelengths of ~350 nm or higher.

**Figure 9:**
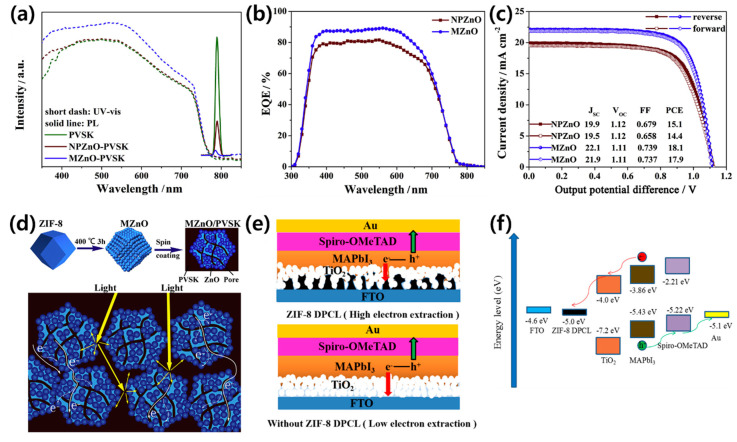
(**a**) The absorption and fluorescence spectra of the perovskite films with and without MZnO. (**b**) External quantum efficiency curves, and (**c**) champion cell performance and hysteresis for PSCs with different electron transport layers (ETLs). (**d**) Schematic of the formation of MZnO and its use as an ETL to enhance light harvesting and electron extraction. Reprinted from [131] with permission. Copyright© 2020, Elsevier. (**e**) Schematic of the perovskite solar cell structures with and without the ZIF-8-derived porous carbon layer, and (**f**) energy level diagram of the ZIF-8 DPCL-based device. Reprinted from [132] with permission. Copyright© 2019, American Chemical Society.

Apart from TiO_2_, ZnO and SnO_2_ are widely used as the ETM [120,121,122,123]. ZnO possesses similar physical properties and energy level positions to TiO_2_ and is an attractive ETM due to its high electron mobility and structural diversity [124,125]. The ZnO layer can be processed at low temperatures, which is advantageous for mass production and application in flexible devices [126,127,128]. However, the PCEs of PSCs based on pure ZnO are only 15–16%, which is significantly lower than those of TiO_2_ or SnO_2_-based PSCs [129,130]. The performance of ZnO is unstable because of the presence of chemical residues in the manufacturing process. Therefore, the quality, charge collection, and recombination rates in ZnO ETMs must be optimized to improve the stability and efficiencies of PCEs. The beneficial properties of MOF-derived porous oxides can assist the optimization of ZnO ETMs by facilitating effective penetration into the perovskite and increasing the contact area between the ETM/perovskite. In this way, MOF-derived porous oxides are excellent candidates to replace mesoporous ETLs. Zhang et al. used MOF-derived ZnO (MZnO) with a dodecahedron porous structure as an ETM [131]. The MZnO inhibited electron extraction and electron-hole recombination rates by quenching the PL intensity, reducing the electron lifetime, increasing the charge recombination resistance, and reducing the density of trap states (Figure 9a,b). The introduction of MZnO increases the PCE (18.1%) compared to pure ZnO-based PSCs (15.1%) by increasing the active electron transport pathways and increasing *J_SC_* and FF. The authors found that the unique shape and large internal pores in MZnO induce higher light absorption densities and effectively improve the optical utilization efficiencies of the PSCs (Figure 9c,d).

ZIF-8 MOF, as an independent, compact ETM in the n–i–p mesoscopic structure, was studied by Zhang et al. (Figure 9e) [132]. They synthesized a ZIF-8 powder and adjusted the nanoparticle size using the solution heating method with polyvinylpyrrolidone (PVP) [133]. The synthesized ZIF-8 MOF was deposited as a ZIF-8 derived porous carbon layer (DPCL) through carbonization and used as the ETL in PSCs. When sunlight enters the PSC, electrons are excited to the perovskite conduction band, subsequently injected into the conduction band of TiO_2_ and carbon, and finally collected at the FTO anode (Figure 9f). Since the work function of FTO is 4.6 eV, ZIF-8 DPCL with a work function of 5 eV is beneficial for electron injection during photoexcitation. Since the electron transport rate can be increased due to the high conductivity and thin ZIF-8 DPCL ETL, the PCE of the PSC improved from 14.25% to 17.32%.

The importance of the ETM is also emphasized in flexible PSCs. PSCs are suitable for use in wearable devices and for building integrated photovoltaic systems due to their excellent mechanical flexibility [134,135,136]. However, since the manufacturing temperature must be low, the high processing temperature of the TiO_2_ layer (>450 °C) makes it difficult to use in flexible PSCs. Ryu et al. succeeded in producing flexible PSCs by applying nanocrystalline MIL-125(Ti) (nTi-MOF) as the ETL at an ambient temperature [137]. From the Tauc plot, they confirmed that the bandgap (3.7 eV) of the nTi-MOF was wider than that of TiO_2_ nanoparticles (3.55 eV). Moreover, the conduction band minimum (CBM) and VBM of the nTi-MOF were −4.12 and 7.82 eV, respectively. The equivalent values for the TiO_2_ nanoparticles were calculated to be −3.98 and −7.53 eV, respectively. Considering that the CBM of the perovskite layer is −3.8 eV, nTi-MOF is suitable as an ETL. The electrical conductivity of nTi-MOF ETL (4.46 × 10^−5^ S cm^−1^) was slightly lower than that of the TiO_2_ ETL (6.38 × 10^−5^ S cm^−1^) and is significantly affected by the film thickness. The conductivity of the nTi-MOF ETL decreased substantially from 4.46 × 10^−5^ to 2.32 × 10^−5^ S cm^−1^ as the film thickness increased from 20 to 60 nm. However, after the deposition of [6,6]-phenyl-C61-butyric acid (PCBM), the conductivity of the nTi-MOF ETL improved considerably to 1.09 × 10^−4^ S cm^−1^. The authors claimed that by coating with PCBM, microcracks are filled in the nTi-MOF ETL and enhanced electrical paths are created, thereby improving the conductivity and inhibiting direct contact between the perovskite and the ITO. This can be directly observed in the photovoltaic performance of the PSCs. The PCE of nTi-MOF rigid PSC without PCBM was 16.41% and 18.94% with PCBM. The PCE of nTi-MOF flexible PSC was 17.43%, which is close to that of the rigid PSC, and the durability was maintained over 700 bending cycles, with a PCE of 15.43%. These results suggest that nTi-MOF has enormous potential in flexible and high-performance PSCs.

### 3.2. MOFs as the Hole Transport Material

In PSCs, the roles of the hole transport material (HTM) are (i) to provide a barrier between the perovskite absorbing layer and the cathode, (ii) to block electron transfer to the cathode, and (iii) consequently inhibit recombination of photoexcited electron-hole pairs at the contact surface. Therefore, the PCEs and long-term stability of PSCs depend heavily on the HTM. To obtain a PSC with high efficiency, the HTM must have excellent thermal and photochemical stability, excellent conductivity, and high hole mobility. In particular, the HTM must have an appropriate energy level for appropriate alignment with the lowest unoccupied molecular orbital of the perovskite to enable effective hole injection from the perovskite to the HTL [138,139]. One of the most common HTMs is 2,2′,7,7′-tetrakis[N,N-di(4-methoxyphenyl)amino]-9,9′-spiro-bifluorene (spiro-OMeTAD). It was initially introduced to PSC for replacing the liquid electrolyte and a high efficiency, up to 9.7%, was achieved [140]. However, the low intrinsic hole mobility and conductivity limit the use of spiro-OMeTAD; therefore, lithium bis(trifluoromethanesulfonyl)imide (Li-TFSI) and tetra-tert-butylpyridine (TBP) were used as additives to improve the conductivity [141,142]. Since Li-TFSI cannot directly oxidize spiro-OMeTAD, a device that controls the degree of oxidation is required [143]. Various additives have been developed that directly oxidize spiro-OMeTAD and enhance the PCE of PSCs [144,145,146,147,148]. However, since the use of an additive involves multiple fabrication processes with low yield or low stability, it is important to develop a material that can be synthesized as an additive with low-cost and simple techniques.

**Figure 10:**
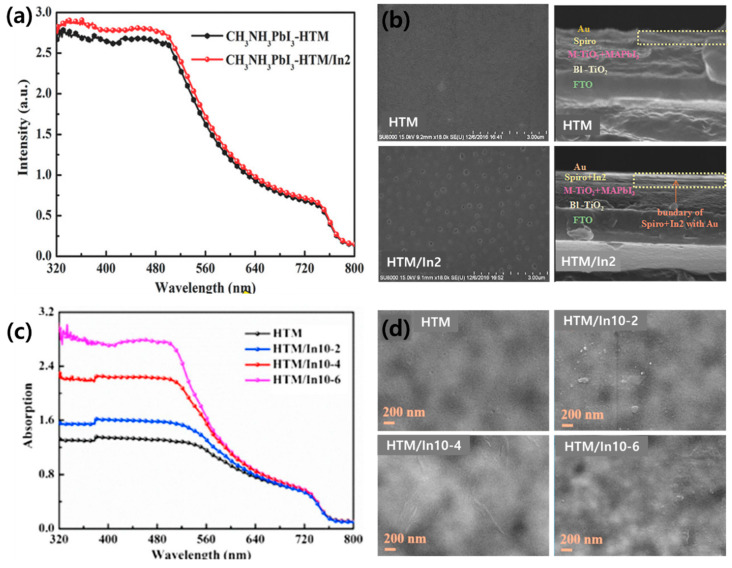
(**a**) UV-vis absorption spectra for the hole transport material (HTM) with and without In2 and (**b**) top view SEM images of films with and without In2. Reprinted from [149] with permission. Copyright© 2017, WILEY. (**c**) The UV-vis absorption spectra of the HTM with different amounts of In10 and (**d**) top view SEM images of HTM, HTM/In10-2, HTM/In10-4, and HTM/In10-6. Reprinted from [152] with permission. Copyright© 2019, Elsevier.

Indium oxide is used as a p-type material in organic electronic devices owing to the excellent conductivity of indium. [In_2_(phen)_3_Cl_6_]·CH_3_CN·2H_2_O (named In2) was introduced by Li et al. as an additive to the HTM through band alignment engineering [149]. Figure 10a shows the UV-vis absorption spectrum of the perovskite film with and without In2 in the spiro-OMeTAD. The shape of the spectra is similar from 320 to 800 nm. However, when In2 was added, the absorption improved from 320 to 540 nm. In the field emission-scanning electron microscope (FE-SEM) image of the HTM and HTM/In2 films, the HTM/In2 film contains few pinholes and is uniformly covered with cubes (Figure 10b). Pinholes cause instability in PSCs because the metal back contact is used as a channel to reach the perovskite [150,151]. Therefore, the addition of In2 provides a dense HTL and serves as a buffer to prevent Au from diffusing throughout the entire PSC structure.

Moreover, In2 plays a role in improving the light absorption of the perovskite, resulting in improved PSC properties (*J_SC_*, *V_OC_*, FF, and PCE). In particular, the PCE improved from 12.8% to 15.8%. Li et al. further synthesized [In_0.5_K(3-qlc)Cl_1.5_(H_2_O)_0.5_]_2n_ (named In10) and employed it as an additive in spiro-OMeTAD [152]. When In10 is added, the color of the spiro-OMeTAD solution changes from light yellow to red-brown, indicating oxidation of the spiro-OMeTAD. As shown in Figure 10c, the addition of In10 improved the UV-vis absorption spectrum between 300 and 500 nm. The overall absorbance increases as the amount of additional In10 increases; however, the film becomes less homogeneous and lumps are formed on the surface that act as new trap sites, thereby causing a decrease in the PSC performance (Figure 10d). An appropriate amount of In10 helps to improve the light response of the PSCs, resulting in a 20% improvement in the PCE compared to cells with no In10.

**Figure 11:**
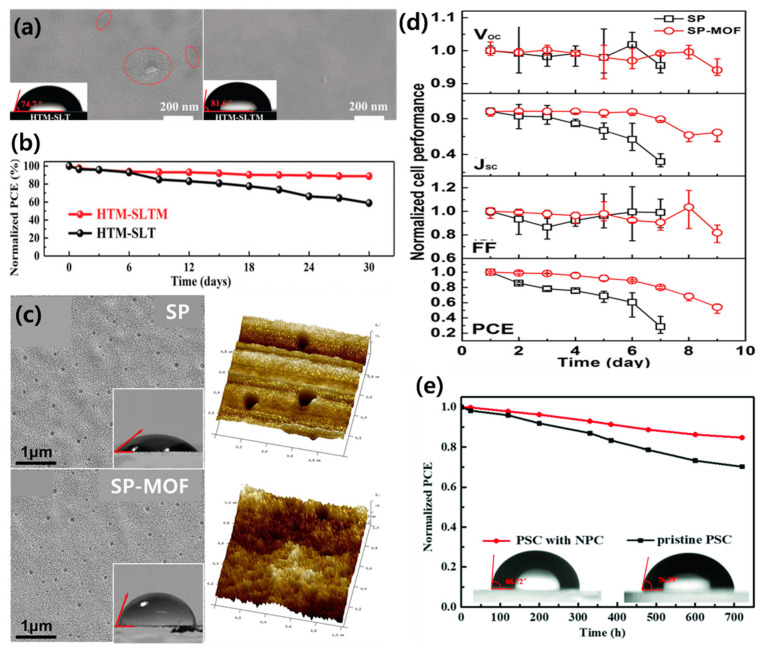
(**a**) SEM images of the HTM-SLT- and HTM-SLTM-based PSCs after long-term stability testing for one month; red ovals highlight cracks (S=spiro-OMeTAD, L=Li-TFSI, T=TBP, M=POM@Cu-BTC; insets: water contact angle test). (**b**) Normalized PCEs over time. Reprinted from [153] with permission. Copyright© 2019, WILEY. (**c**) SEM surface images (insets: hydrophily) and 3D AFM profiles for SP and SP-MOF samples. (**d**) The typical *J–V* curves of PSCs with SP and SP-MOF HTLs over time, in a wet environment. Reprinted from [160] with permission. Copyright© 2019, Elsevier. (**e**) Stability measurements for the pristine PSCs and PSCs with NPC (insets: water contact angles). Reprinted from [161] with permission. Copyright© 2019, WILEY.

Dong et al. used polyoxometalate@MOF (POM@MOF; [Cu_2_(BTC)_4/3_(H_2_O)_2_]_6_[H_3_PMo_12_O_40_]_2_ or POM@Cu-BTC) as an HTM dopant [153]. POM@MOF can control the oxidation of spiro-OMeTAD and improve the stability of the HTL. POMs are metal oxo-clusters and have high electron affinity and oxidation potential. The nanoporous structure of the Cu-BTC MOF and solid-state nanoparticles improves the water stability of the HTL, allowing an improvement in the long-term performance of the PSC. Figure 11a shows an SEM image of the PSC with and without the POM@MOF after a stability test over one month. In the PSC without the POM@MOF, obvious cracks were formed; however, the film containing the POM@MOF maintained a uniform surface coverage. The stability of the perovskite film is directly related to the PSC performance. By doping with the POM@MOF, the PCE of the PSC improved from 20.21% to 21.44%, and, remarkably, it maintained approximately 90% of the initial PCE value after long-term storage in an ambient environment (Figure 11b).

Inorganic HTMs are studied because spiro-OMeTAD has a relatively low carrier transferring ability and poor moisture resistance. Metal oxides, such as NiO, Cu_2_O, MoO_3_, and V_OX_, were used as the HTM, preventing moisture from penetrating through the device, and improving the stability [154,155]. In particular, NiO is attracting significant attention as a p-type inorganic HTM due to its low processing cost, excellent hole extraction, exceptional thermal and chemical stability, and deep valence band level. However, due to the low conductivity of NiO and surface defects, the efficiency of NiO-based PSCs is still lower than that of spiro-OMeTAD-based PSCs. The low conductivity leads to hole accumulation near the perovskite interface, increases the likelihood of electron-hole recombination, and reduces the charge collection efficiency [156]. Hazeghi et al. improved the efficiency of NiO-based PSCs using CuO with an electrical conductivity higher than NiO, improving hole extraction, reducing the trap density in NiO, and suppressing the recombination rate at the perovskite/HTL interface [157]. The PCE of PSCs using the NiO HTL was 8.58%, whereas that of the PSCs using core-shell CuO@NiO as the HTL was 10.11%. PSCs based on the CuO@NiO HTL showed lower PCEs than PSCs based on spiro-OMeTAD; however, excellent long-term stability was achieved. PSCs based on NiO and CuO@NiO HTLs maintain 52% and 60%, or more, of the initial efficiency, respectively, after 1920 h (80 days), whereas PSCs based on the spiro-OMeTAD HTL under the same conditions degrade after 1248 h (52 days). Only 31.74% of the initial efficiency was maintained. These results suggest that the replacement of spiro-OMeTAD with a NiO HTM using MOFs can produce promising PSCs with superior long-term stability.

Metal doping is another effective way to optimize the HTL performance in PSCs. Among the various dopants employed, 2D nanomaterials have been attracting attention as materials with high electrical conductivity due to their unique properties such as special planar motion and strong covalent bonding [158]. In particular, 2D MOFs of Cu-BHT (BHT = benzenehexathiol) showed high conductivity and transmittance and can replace ITO [159]. Therefore, 2D MOFs are sufficiently qualified as a conductive material, and many studies have been conducted, wherein a 2D MOF is doped into the HTM. Huang et al. successfully synthesized 2D Pb-MOF hexagonal sheets and mixed them with the spiro-OMeTAD solution for use as a HTM in PSCs [160]. The difference in surface characteristics due to the addition of 2D Pb-MOF cannot be distinguished in the SEM images; however, the root mean square (RMS) roughness values of the samples are significantly reduced with (0.765) and without (1.58) the 2D Pb-MOF (Figure 11c). The hydrophobicity of the pristine HTL and the Pb-MOF HTL were investigated, and wetting angles were measured to be 39° and 71°, respectively. In general, the larger the RMS, the greater the wetting angle. It can be inferred that the high hydrophobicity of the Pb-MOF sample is due to the composition of the material rather than its roughness. The initial efficiency of the PSC without Pb-MOF was 10.53%, and 28% of the initial value remained after 9 days, whereas the PSC with Pb-MOF retained 54% of the initial efficiency (13.17%) after 9 days (Figure 11d). Therefore, through these results, 2D Pb-MOF increases PSC performance through improved stability and PCE when a Pb-MOF HTM is used. Another 2D MOF material for improving the stability of PSCs is 2D graphitic N-rich porous carbon (NPC), used as an auxiliary additive [161]. NPC optimizes the film quality by reducing the aggregation and defects in lithium salts used as additives to spiro-OMeTAD, allowing for fast hole extraction and migration. Furthermore, the inherent porosity and hydrophobicity of NPC improve the stability of PSC by limiting the permeation of Li^+^ and the anode metals and can prevent moisture from entering the HTL and perovskite layers (Figure 11e).

### 3.3. MOFs as the Interlayer

The perovskite film itself also influences the stability and reproducibility of PSC photovoltaic performance [162]. From this viewpoint, research has been conducted to obtain a high-quality perovskite film with high crystallinity [163,164,165,166]. High-quality perovskite films can be obtained by manipulating the growth and nucleation of the perovskites, and this approach can be divided into two aspects. One aspect is to obtain an extremely uniform perovskite thin film with high crystallinity by solvent engineering upon deposition of a perovskite precursor solution [167]. Another aspect is to improve the performance of the device by controlling the crystal growth and improving the film quality through interfacial engineering between the perovskite and ETL. Therefore, interlayers play a vital role in the efficiency of PSCs and in improving stability [168]. Sb_2_S_3_ [169], plasmonic Au nanoparticles [170], MgO [171], and Au@SiO_2_ [172] are used as interlayers between the perovskite and TiO_2_ layers to reduce hysteresis in the PSC and to improve the stability of devices. However, apart from Sb_2_S_3_, the syntheses of these materials requires a long time and a high temperature. In contrast, MOFs are easily synthesized and their bandgap can be adjusted. Therefore, the application of MOFs as the interlayer is also a valuable tool in PSCs.

**Figure 12:**
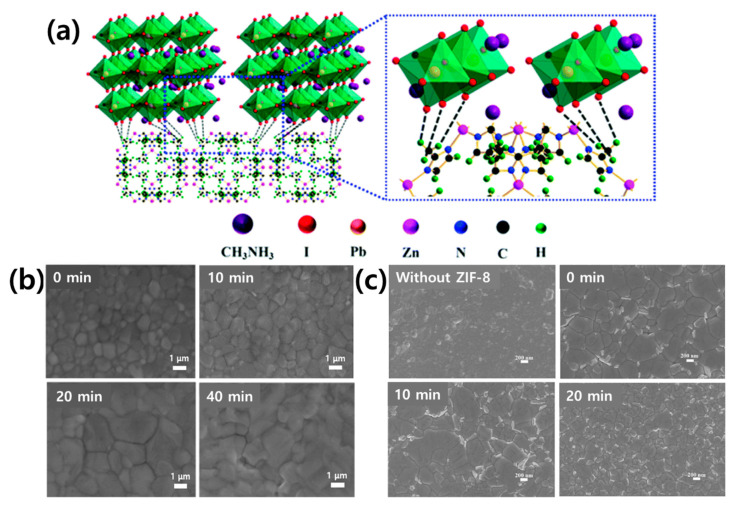
(**a**) Schematic of two neighboring grain structures crosslinked by the methyl groups in ZIF-8. Reprinted from [173] with permission of The Royal Society of Chemistry. (**b**) SEM images of the MAPbI_3_-based layer formed on the surface of mp-TiO_2_ with different ZIF-8 coating times: 0, 10, 20, and 40 min. Reprinted from [174] with permission. Copyright© 2015, WILEY. (**c**) SEM images of the perovskite films formed on c-TiO_2_ and c-TiO_2_/ZIF-8 layers with different ZIF-8 synthesis times; 0, 10, and 20 min followed by ultrasonic vibration for 3 min. Reprinted from [175] with permission. Copyright© 2020, American Chemical Society.

The Wei group first used ZIF-8 as an interlayer between the mesoporous (mp)-TiO_2_ and perovskite layers [173]. Figure 12a shows a schematic of the interaction between the ZIF-8 layer and perovskite film. When the ZIF-8 layer is coated on the surface of the mp-TiO_2_ film, it can then act as an additional scaffold to support crystal growth in the early stages of crystallization of the subsequently deposited perovskite film [174]. In the crystal structure of ZIF-8, the methyl group can form a hydrogen bond with the halide anion of the perovskite, thereby improving the cohesive force between the perovskite film and substrate. Therefore, the optimal amount of ZIF-8 can be effectively combined with adjacent perovskite grains to reduce the perovskite particle size and roughness and to form a high-quality light-harvesting layer on the surface of the mp-TiO_2_ film (Figure 12b).

The mp-TiO_2_/ZIF-8 PSC exhibited a PCE of 16.99%, higher than that exhibited by mp-TiO_2_ PSC (14.75%). It was confirmed that the ZIF-8 interlayer inhibits the recombination of photogenerated carriers at the interface and improves charge extraction. Similarly, ZIF-8 was used in place of the mp-TiO_2_ layer; the Eslamian group produced a PSC displaying a PCE of 16.8% using ZIF-8 as an interlayer between the compact(cp)-TiO_2_ and perovskite layers [175]. As shown in Figure 12c, the grain size of the perovskite significantly increased when ZIF-8 was used as an interlayer. The larger particle size due to the ZIF-8 interlayer reduces the number of charge carriers trapped by defects, and the band alignment between the perovskite and ZIF-8 creates an energy barrier to protect the exciton from surface defect traps. As can be seen, the mp-TiO_2_ can be replaced with the easily synthesized ZIF-8.

Unlike other groups that used MOFs as an interlayer between the ETL and perovskite layers, Nguyen et al. applied the MOF between the perovskite layer of the n–i–p structure and HTL [176]. The interlayer minimizes the energy losses at the perovskite/HTL interface and can adjust the energy level mismatch between the two layers. Electrons moving from the conduction band of the perovskite to the valance band of the HTL can also be suppressed [177,178]. The effect of NiO as an interlayer between the perovskite and spiro-OMeTAD layers was demonstrated [179]. NiO@C was synthesized and used to improve the efficacy of the NiO interlayer. The NiO@C interlayer was found to delay the recombination of electron-hole pairs in the active layer and reduce the charge transport resistance, consequently increasing the PCE of the PSC from 13.79% to 15.78%.

### 3.4. Hybrid Perovskite-MOFs

As we continue to emphasize, the most important point in PSCs research is improving the efficiency and stability. The stability of PSCs is primarily related to the inherent defects present in the perovskite layer. PSCs are vulnerable to moisture as the lattice collapses due to the formation of hydrogen bonds between H_2_O and perovskite constituent ions [180,181]. Moreover, intense thermal/photo stresses cause iodide oxidation, resulting in the formation of I_2_ and volatilization of CH_3_NH_2_, leading to material degradation [181,182]. In the previous section, we described a study that improved the particle size in the perovskite using MOFs as a porous scaffold interlayer [173]. Based on this study, another investigation was conducted to improve the performance and stability of devices by mixing perovskites and MOFs to form a hybrid perovskite-MOF (P-MOF) PSC.

**Figure 13:**
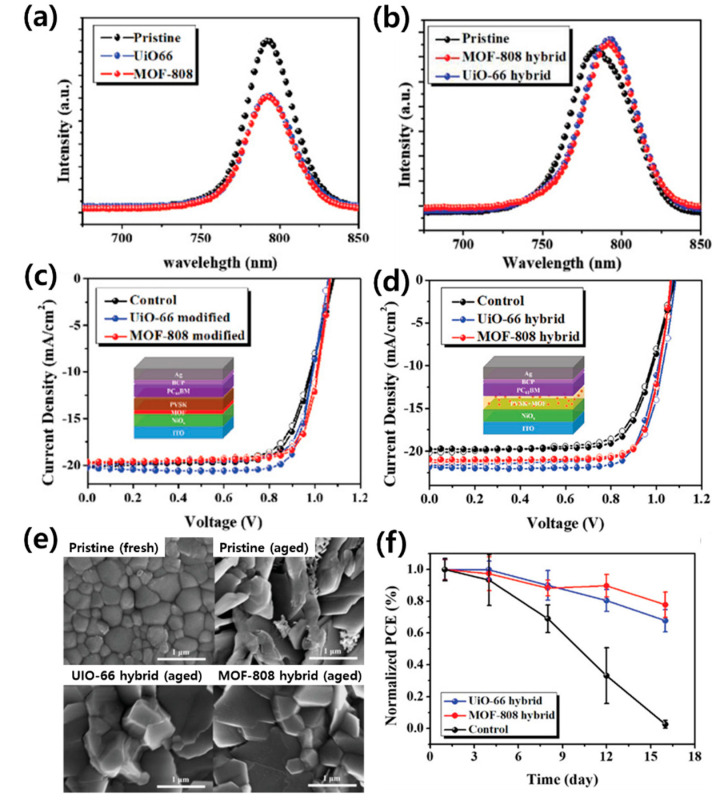
Photoluminescence spectra of (**a**) bilayer MOF/perovskite films, and (**b**) hybrid P-MOF films. The *J–V* curves of (**c**) the PSC employing MOF as a bilayer and (**d**) the hybrid P-MOF PSC. (**e**) SEM images of the films after 30 days of aging. (**f**) The PCEs of the fabricated devices as a function of storage time in ambient air (25 °C and RH: 60 ± 5%). Reprinted from [183] with permission.

Studies using two types of Zr-MOF (MOF-808 and UiO-66) with different physical properties (pore size and tunnel structure) separately as the interlayer and hybrid P-MOF have been reported [183]. Figure 13a,b show the PL spectra of the perovskite layer with a MOF interlayer and hybrid P-MOF, respectively. When the MOF was used as an interlayer, charge transfer was promoted at the interface between the perovskite and the MOF, and PL quenching occurred. In contrast, the PL intensity of the hybrid film was higher than that of the pristine perovskite film, and this enhancement implies defect passivation induced by the MOF. The PCE of PSCs using Zr-MOF as the interlayer and hybrid P-MOF was improved than that of the control device, and that of UiO-66 based PSC was more improved than that of the MOF-808-based PSC (Figure 13c,d). Direct hybridization of the MOF and the perovskite appears to slightly inhibit the growth of perovskite grains; however, the overall impact is small (Figure 13e). The hybrid P-MOF structure allows the passivation of defects and enhances the resistance of the film to moisture penetration, thereby improving the PSC efficiency and stability (Figure 13f).

In general, Zr-based MOFs require a complicated synthesis at high temperatures; therefore, there are some difficulties in the manufacturing process [184,185,186,187,188]. In contrast, a study using In-based MOF (In2) with relatively flexible synthetic conditions and adequate stability as the HTM was previously described [149]. The charge transfer was accelerated by band alignment and the optical response of the PSCs also improved. In2 was likewise applied to hybrid P-MOFs [189]. The authors improved the performance of PSCs by mixing In2 with the PbI_2_ precursor solution. The large conjugated In2 system favors charge transfer, and as such, In2 can be integrated into the PbI_2_ precursor solutions. Studies have shown that unpassivated Pb^2+^ (due to loss of methylamine) is detrimental to PSC performance [190]. Therefore, the addition of In2 changed partial Pb^2+^ to Pb, thereby solving the problem of performance degradation caused by excessive Pb^2+^. When the amount of In2 was optimized, the PCE of PSCs improved significantly from 15.41% to 17.15%. Zhou et al. synthesized microporous indium-based MOF [In_12_O(OH)_16_(H_2_O)_5_(BTC)_6_]_n_ (In-BTC) nanocrystals under mild conditions, and through mixing with perovskites, produced PSCs [191]. In-BTC improves the morphology and crystallinity of the perovskite, reducing the grain boundaries and defects in the film. Therefore, In-BTC provides improved PCE (19.63%) compared to the pristine PSC (18.19%), and more than 80% of the initial PCE is retained after 12 days, demonstrating the long-term stability (pristine PSC retained 35.4% of the initial PCE).

To summarize MOF-based PSCs, the long-term stability and PCE have improved owing to the excellent chemical and thermal stability of the MOFs (Table 2). MOFs were applied in various forms, such as the HTM, ETM, interlayer, and hybrid P-MOFs. The applied forms and MOF materials are different, but show similar results, and the role of MOFs in PSCs can be summarized as follows [192]: (i) they can improve the quality and crystallinity of the perovskite films; (ii) they can improve charge transfer and suppress charge recombination; and (iii) they improve device stability. The results of these studies provide important information for future designs of PSCs with high efficiency and long-term stability.

## 4. Summary

In summary, the biggest problem to be solved for the commercialization of third generation solar cells is device stability. In particular, PSCs, despite exhibiting a high PCE similar to single-crystal silicon solar cells, still lack long-term stability performance. Therefore, research focusing on improving the performance and stability of PSCs is ongoing. Herein, MOF materials are introduced to improve performance. MOFs have unique properties and are used in a wide range of applications. The advantage of facile bandgap adjustment by changing the components during the simple syntheses makes MOFs attractive materials for PSCs. MOFs and MOF-derived materials can be placed in various positions in the PSCs, improving the quality of the perovskite film, enhancing charge transfer, and inhibiting charge recombination. Therefore, MOF and MOF-derived materials contribute significantly to improving the efficiency and stability of PSCs. However, the exact mechanisms for these improvements have not been confirmed, and an insightful investigation is needed using various MOF materials. Since MOFs can be expanded and used as a MOF-derived material, they are expected to play a pivotal role in improving PSC performance.

## Figures and Tables

**Table 1:** Photovoltaic performances of DSSCs with MOFs applied as the photoanode and counter electrode.

	MOF Material	V_OC_ (V)	J_SC_ (mA/cm^2^)	FF	PCE (%)	Ref
Photoanode	ZIF-8	0.753	10.28	0.69	5.34	[82]
	MIL-125	0.85	10.9	0.69	6.4	[83]
	Zn	0.68	6.22	0.55	2.34	[84]
	NGE/NiO	0.76	19.04	0.67	9.75	[85]
	ZIF-8	0.66	8.13	0.68	3.67	[86]
	Ni-MOF	0.624	27.32	0.516	8.84	[87]
Counter electrode	MOS_2_@Co_3_S_4_	0.782	16.21	0.62	7.86	[88]
	CoSe_2_-NC@Co-FeSe_2_	0.806	17.9	0.66	9.61	[89]
	CoNi@CNTs	0.76	18.3	0.65	9.04	[90]
	ZIF-8/GO	0.77	15.25	0.69	8.2	[91]

**Table 2:** Summary of the photovoltaic performance of MOF-derived PSCs discussed in the text.

	MOF Material	V_OC_ (V)	J_SC_ (mA/cm^2^)	FF	PCE (%)	Ref
ETM	Co-doped Ti-MOF	1.027	24.078	0.649	15.75	[118]
	ZIF-8	0.936	21.6	0.62	12.4	[119]
	ZIF-8 derived ZnO	1.11	22.1	0.739	18.1	[131]
	ZIF-8 derived porous carbon	1.06	22.13	0.72	17.32	[132]
	MIL-125(Ti)	1.01	22.81	0.72	16.56	[136]
	MIL-125(Ti)/PCBM	1.082	23.18	0.755	18.94	[137]
HTM	In2	1.01	21.03	0.74	15.8	[149]
	In10	1.00	24.3	0.70	17.0	[152]
	POM@Cu-BTC	1.11	23.90	0.80	21.44	[153]
	CuO@NiO	0.91	21.80	0.51	10.11	[157]
	2D Pb-MOF	1.00	19.57	67.30	13.17	[160]
	2D graphite NPC	1.06	23.51	0.76	18.51	[161]
Interlayer	ZIF-8	1.02	22.82	0.73	16.99	[173]
	ZIF-8 (scaffold layer)	1.23	21.8	0.59	16.8	[175]
	NiO@C	1.018	22.394	0.69	15.78	[176]
	MOF-808	1.068	19.64	0.79	16.55	[183]
	UiO-66	1.067	20.25	0.78	17.01	[183]
Hybrid Perovskite-MOF	MOF-808	1.062	21.01	0.80	17.81	[183]
	UiO-66	1.072	21.85	0.77	18.01	[183]
	In2	1.04	23.18	0.71	17.15	[189]
	In-BTC	1.10	22.99	0.77	19.63	[191]
